# Advanced Access in Primary Healthcare and Its Effects on Emergency Department Utilization: A Rapid Review

**DOI:** 10.3390/healthcare13121430

**Published:** 2025-06-15

**Authors:** Rafael Tannure, Salma Sarkis, Amanda Peres, Juliana de Souza Lapa, Lígia Villela Rodrigues, Italo Landim, Ciro Martins Gomes, Katia Crestine Poças, Henry Maia Peixoto, Sandro Rogério Rodrigues Batista, Rodolfo Deusdará

**Affiliations:** 1 Faculty of Medicine, University Center of Brasília, Brasília 70790-075, Brazil; salmasarkissimao@gmail.com (S.S.); amandabarbosaperes@gmail.com (A.P.); 2 Secretaria de Estado de Saúde do Distrito Federal, Brasília 70719-040, Brazil; ligiavrodrigues@gmail.com (L.V.R.); italolandimm@gmail.com (I.L.); 3 Faculty of Medicine, University of Brasília, Brasília 70910-900, Brazil; julianaslapa@gmail.com (J.d.S.L.); ciromgomes@gmail.com (C.M.G.); katiacrestine@gmail.com (K.C.P.); henrymaiap9@gmail.com (H.M.P.); 4 Postgraduate Program in Tropical Medicine, Faculty of Medicine, University of Brasília, Brasília 70910-900, Brazil; 5 Postgraduate Program in Medical Sciences, Faculty of Medicine, University of Brasília, Brasília 70910-900, Brazil; sandrorbatista@gmail.com; 6 National Institute of Science and Technology for Health Technology Assessment (IATS), Porto Alegre 90035-903, Brazil; 7 Epidemiology Laboratory, Center for Tropical Medicine, Faculty of Medicine, University of Brasília, Brasília 70910-900, Brazil

**Keywords:** primary healthcare, emergency department, hospital, health services accessibility

## Abstract

**Background:** The advanced access (AA) scheduling model in primary healthcare (PHC) may reduce unnecessary visits to the emergency department (ED). However, evidence of this effect remains uncertain and limited. **Objective:** To evaluate whether the adoption of AA models in PHC may reduce ED visits, when compared to the traditional model. **Methods:** A rapid review of the literature according to the World Health Organization’s guidelines was performed, using two databases (PubMed and Lilacs) with articles from 1980 to 2023. **Results:** A total of 1286 articles were found according to our search. Of them, 1245 were excluded based on their titles, most of them due to not evaluating advanced accesses as an intervention. Of the remaining 41 articles, many did not evaluate ED visits as an outcome, nor did they have the criteria of inclusion. Eight articles evaluated ED visits as an outcome and had inclusion criteria. Five articles were included and three found an association between the adoption of advanced access in PHC and a reduction in ED visits. **Conclusion:** This review shows that the adoption of AA in PHC may reduce ED visits. However, it is essential to carry out new studies to understand the relationship between the adoption of AA in PHC and its outcomes in universal healthcare systems.

## 1. Introduction

Primary healthcare (PHC) is the first level of a health system and, proprietarily, must provide access to treatment for all health needs and problems, give attention to patients over time, care for all conditions except very uncommon or rare conditions, and coordinate or integrate care provided in other levels of the health system [1,2,3,4]. It is characterized by a comprehensive and integrated approach, organizing and rationalizing the use of health resources, as well as addressing the most common problems in the community by offering prevention, treatment, and rehabilitation services to maximize health and well-being [5,6].

To achieve these objectives, PHC must deploy a scheduling strategy as a crucial organizational component that allows individuals to access primary care services. Evidence shows that prolonged wait times for PHC appointments were associated with high risks for morbidity and mortality [1,7,8,9], and reducing wait times for mental health problems has improved access to mental healthcare and the efficient use of resources [10,11].

Frequently, PHC scheduling is based on the conventional approach. According to this, the vast majority of consultations, known as routines, are scheduled, leaving limited opportunity for spontaneous demand consultations [12,13]. Thus, it is common to observe a higher number of emergency department (ED) visits as an alternative for patients with acute complaints [14,15,16,17], and, consequently, problems associated with primary healthcare as the first contact for care are common [18,19].

In this context, advanced access (AA) is a strategic technology that may expand access and improve quality delivery in PHC. The model, based on the pioneering formulations of Murray and Tantau [8], has the availability of healthcare professionals to meet demand, with the central rule being “do today’s work today” as its core principle. AA models must adhere to five basic principles: (1) Balancing the supply of and demand for appointments; (2) reducing appointment backlog by eliminating waiting lists; (3) revising the appointment scheduling system to allow for short-term planning (two to three weeks); (4) enhancing professional integration by optimizing roles and directing patients to the appropriate provider; and (5) developing contingency plans to accommodate demand increases [7,20,21].

Thus, AA is characterized by a greater proportion of places available for spontaneous demand services (greater than 65% of available places), with a small number of places reserved for routine care. Furthermore, in this model, the patient must receive care, ideally within 48 h of beginning to seek care [7,8,9]. Accordingly, the model offers an increased number of available appointments for unplanned visits (walk-ins, same-day visits) and may reduce both wait times for appointments and patient no-show rates [20,21].

Despite the original formulation, the implementation of AA may not occur uniformly across different settings. Evidence of the effects of AA on ED visits [16,17] and other clinical outcomes remains uncertain and limited [1,22,23,24,25]. To evaluate the outcomes of adopting advanced access practices, especially in the emergency department, this work carried out a rapid review of the literature.

## 2. Materials and Methods

This rapid literature review was carried out according to the guidelines of the World Health Organization (WHO)’s practical guide for rapid reviews. It took place between March 2023 and July 2023, following the steps described below in their respective sub-items [26,27].

The first stage of the review process was the elaboration of the research protocol, with the definition of the research question and the choice of terms for searching the databases. Subsequently, literature research, screening and selection of studies, data extraction, and quality assessment of studies were carried out. All steps that constitute the selection of studies, quality assessment, and data extraction were carried out by a reviewer supervised by a more experienced researcher to resolve doubts and guide the process.

### 2.1. Preparation of the Research Question

The elaboration of the question for this rapid review aims to guide the search for scientific evidence regarding the benefits of AA in PHC compared to the traditional scheduling model. To structure a research question, most authors use the PICO format (population, intervention, comparator, and outcomes). The structure of our research question is found in Table 1.

Therefore, the structured research question for this course conclusion work is as follows: For PHC patients who sought care in an emergency department, does the adoption of advanced access reduce the use of emergency services compared to the traditional emergency department model?

### 2.2. Database Search

The search for evidence was carried out through a rapid review of the literature. For this, two databases were used: PubMed and Lilacs. The articles included in the review dated from the period between January 1980 and July 2023, with search strategies adapted for the different databases.

**Figure 1:**
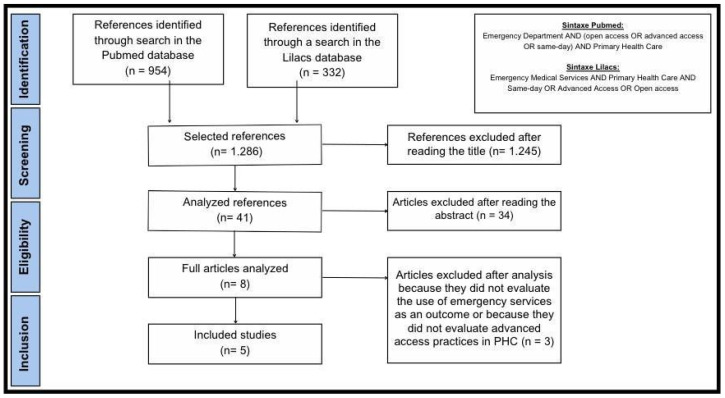
Article selection flowchart.

In the PubMed database, we chose to use the terms open access, advanced access, and same-day, all joined by the Boolean operator “OR”, associated with the terms MESH: Emergency Departments and Primary Healthcare, using the operator Boolean “AND” (Figure 1).

In the Lilacs database, the search was carried out using the terms DECS: Emergency Medical Services and Primary Healthcare, both linked by the Boolean operator “AND”, associated with the terms same-day, advanced access, and open access, all linked by the Boolean operator “OR” (Figure 1).

### 2.3. Study Selection Criteria

To select the articles, the titles/abstracts that met the research inclusion criteria were initially reviewed by two independent reviewers, and, subsequently, in those for which there was doubt regarding the inclusion criteria, their abstracts were analyzed by a third reviewer. Finally, the articles selected after this first phase were read in full, evaluating the inclusion and exclusion criteria.

As this was a rapid review, the selection of texts that would be part of the study was not carried out in pairs.

For the inclusion of articles, the following criteria were used:

1. Articles that evaluate some form of advanced access as an intervention in primary healthcare.
2. Articles that evaluated the use of emergency services as an outcome.

The following criteria were used to exclude articles:

1. Articles that do not evaluate the use of urgent and emergency services as an outcome.
2. Articles that evaluate as the main space of care a space other than primary healthcare.
3. Articles classified as systematic reviews.

### 2.4. Quality Assessment of Selected Studies

Quality assessment of cohort studies was carried out using the Newcastle-Ottawa scale. This scale is based on a star rating system with a maximum of nine stars for the lowest risk of bias. The evaluation takes place in three areas: (1) selection of study groups (four stars); (2) comparability of groups (two stars); and (3) investigation of exposure and results (three stars). A study is considered high quality when it receives 7 or more stars and moderate when it receives 5 or 6 stars [28,29].

The quality assessment of the other studies was carried out using the quality assessment tool for before–after (pre–post) studies with no control group and the quality assessment tool for observational Cohort and Cross-Sectional Studies made available online by the National Institutes of Health [30] (https://www.nhlbi.nih.gov/health-topics/study-quality-assessment-tools) (accessed on 10 January 2025), whose critical points found are listed in the results. Two independent reviewers participated in this quality assessment and a consensual decision was made.

## 3. Results

Of the 1286 articles found, 1245 were excluded based on the title, most of them because they did not involve advanced access as an intervention in primary healthcare. Of the remaining 41 articles, many did not evaluate the use of emergency services as an outcome, nor did they have the criteria of inclusion, leaving eight articles that were read in full, five of which were accepted for evaluation in Figure 1 [31,32,33,34,35].

The identification and selection of studies, which were carried out according to the inclusion and exclusion criteria described, are presented in the flowchart in Figure 1.

Regarding the assessment of the quality of the studies, carried out in accordance with the NOS, the rating of the cohort studies is listed in Table 2. It can be noted that two of the studies are of high quality (7 or more stars) and one is of moderate quality.

In relation to the other two studies, the critical points are predominantly found in exposure measures, which were not clearly established, reliable, or implemented consistently across all study participants (Table 3).

### Summary of Results

Of the five articles found that met inclusion and exclusion criteria, two found no association between AA and the number of ED visits, while three of them found an association between AA in PHC and reduced ED visits.

It is interesting to note that the design of the studies was quite heterogeneous, with some evaluating economic indicators and others evaluating outcomes by the number of ED visits. Exposure measurements were also heterogeneous, demonstrating variability in the application of AA principles.

Despite such heterogeneity, most of the studies included in this research evaluated PHC services linked to health plans in the United States and Canada. The qualitative synthesis of the studies is found in Table 4 and summarized in the next section of this article.

Glass and collaborators [34] conducted a study in California with California Steel Industries (CSI) employees insured by Kaiser Permanente (KP) between 2007 and 2014. In January 2010, both employees and their dependents began to have access to Family Health at the company itself, thus reducing access barriers by reducing travel time, providing a guarantee of same-day care, and having no requirement for co-participation.

To comparatively evaluate the data, a control group was established in which patients insured by Kaiser Permanente, but who did not belong to CSI, were included. For these patients, care was provided at Kaiser Permanente-affiliated medical centers in the Inland Empire, where patients in the intervention group were cared for before 2010.

As a result, the study found a 43% drop in the use of emergency care in the intervention group, while in the use of the emergency department, no association was observed, despite there being a 4% drop in use in the intervention group and an 8% increase in the control group (*p* = 0.017). In the same study, the use of the emergency department was also compared between CSI employees insured by KP and their dependents, verifying a 30% reduction in the group of employees and an increase in ED visits of 11% in the group of dependents. The two groups differed in terms of distance to the PHC service.

In 2004, in a study by Solberg and collaborators [35], similar results were found. The study included patients with diabetes, chronic cardiovascular disease, and depression over the age of 18, identified in the health plan databases. The use of health services was evaluated in 1999 and 2001, the period before and after the implementation of advanced access to primary care services. As a result, there was a 1/3 drop in the need for urgent care; however, the change was slight in the use of emergency services.

Hudec and colleagues [33] found significant results regarding emergency department use. In their study, economic indicators for the months of April, May, and June of the year before and after the implementation of advanced access in a health unit were included. Based on these economic indicators, a 28% reduction in non-urgent and less urgent patients in the emergency department was observed.

In another study carried out with patients who consulted at least twice in one of the 22 primary care clinics of the Veterans Health Administration (VHA) in 2009, information was obtained regarding the patients’ diagnoses and their use of health services [33]. Another aspect measured was the percentage of patients receiving access on the first day in each clinic. It was observed that in clinics with low same-day access (less than 40% of patients), there was a significantly higher rate of emergency visits compared to clinics with high same-day access (*p* < 0.001).

Finally, in a study carried out in Alberta, Canada [32], access to primary healthcare was measured by the third-next appointment available (TNAA). A TNAA value of 0 indicates that the patient can receive same-day care, while a value of 14 indicates that the patient must wait approximately 14 days for an appointment.

In this study, the TNAA values of each professional were recorded between January 2009 and January 2017. The objective of the study was to associate the TNAA values directly with the patients’ activity, obtained from the National Ambulatory Care Reporting System. From the study, it was possible to identify that professionals who improved their TNAA over a period of one year achieved a reduction in the use of the emergency department compared to professionals with stable TNAA.

## 4. Discussion

The effects of adopting AA in PHC and its outcomes on ED visits present conflicting results in the literature. A study with analysis of three systematic reviews shows no association between AA and ED visits by diabetic patients and patients with cardiovascular diseases after the adoption of AA [22]. Rose and colleagues [36] found that AA, in general, does not appear to be a robust method for improving clinical outcomes, and a review carried out by Rivas [37] found data suggesting that the adoption of AA practices reduces the demand for ED visits

In this work, of the five articles selected, three presented moderate to high quality and indicated that the implementation of AA reduced the demand for ED [32,33,35]. The others, also with moderate to high quality, despite not finding a reduction in the use of emergency services, achieved a reduction in emergency visits [31,34]. Although the last two studies did not find a correlation between AA in PHC and decreased ED demand, one of them explicitly points out in its discussion that it did not account for national trends in ED use, which could be a confounding factor in the analysis given a national trend of increased ED use. It is important to note that the exposure assessments in the studies selected for this analysis varied, and only two of them employed TNAA, the most reliable indicator to assess this outcome. Thus, enhanced access was achieved as TNAA improved [22,35].

In the other studies, the measures of AA varied: the percentage of patients receiving appointments with less than one day in PHC [32] and gained advanced access due to the fact that there were only 12 medical visits per day at their medical center and a high patient satisfaction rate for wait times [34]. This fact, in itself, implies the interpretation of the studies, after all, since exposure measures were divergent, it is possible to infer that the interpretation of advanced access and its real application varied between the studies. The same variation was observed in the assessment of outcomes, with some studies observing the outcome by economic indicators, others by classification in screening in the ED, and others by reviewing diagnoses made in the ED and classifying them into categories. As an example, an important study conducted in England that evaluated access to PHC and ED visits [38] utilized the following outcomes: percentage of patients who succeeded in seeing a GP within ≤2 weekdays on their last attempt (past 6 months), percentage able to book a GP appointment more than 2 weekdays in advance, the percentage who found it “very/fairly easy” to contact a GP by phone, and the percentage who saw their preferred GP “always/almost always/a lot of the time”. Even when considered with important indicators for evaluating first-contact access in PHC, the authors did not use the AA as an exposure variable and because of that, the study was not included in our analysis.

The heterogeneity of exposure and outcome measures probably arises from the operational and methodological difficulties of carrying out studies with the aim of evaluating the research question of this work, which may, for example, justify the absence of randomized clinical trials on the topic [26,27]. Furthermore, the lack of standardization of the AA concept and exposure and outcome measures impairs comparability between studies, making it difficult to consistently observe results in different scenarios [39].

In other reviews related to this subject evaluating other outcomes of AA in PHC, the results were varied. Some studies [22,36] conclude that advanced access to PHC has no impact on the use of emergency services for patients with diabetes and cardiovascular diseases; Rivas [37] concludes that additional studies on the topic are needed.

Despite this, our review differs from these previous reviews because we had the specific objective to analyze the effects of AA on ED visits. It is important to note that although our work was structured as a rapid review, more studies were included in comparison to other reviews. Like most rapid reviews, this work presented limitations related to the method, such as limited searches, selection, and extraction. Likewise, the limitation of the bases used and the syntax may impact the scope of this rapid review. This should be taken into account when interpreting the results of this review [26,27].

In order to reduce such limitations, typical of a rapid review, some measures were adopted based on previous recommendations [40], namely the choice of comprehensive databases, supervision of the review stages by experienced researchers, and holding meetings to standardize concepts and organize the execution of all stages. Furthermore, for the reality of PHC, this rapid review was able to provide a rapid response based on good evidence. In this way, rapid review, recommended by the World Health Organization to strengthen health policies and systems, becomes a feasible method to gather evidence and support rapid decision making on organizational and clinical issues, preventing decisions from being made based on less robust evidence such as expert opinion or the results of a single small study [26,27].

As a limitation, it is noteworthy that most of the studies included were carried out among patients insured by private health plans in the United States and Canada. No studies were found in a scenario of public and universal health systems, such as the Brazilian one. Thus, the applicability of the conclusions of this review to the reality of Brazil becomes limited. Finally, the studies selected for our review were of moderate to high quality, as reported in the results. Therefore, the evidence is good to support the findings of this rapid review.

## 5. Conclusions

AA is an intervention that aims to reduce the wait time for an appointment with a healthcare professional. Ideally, by definition, the patient should obtain care within a maximum of 48 h. Despite this, there is great variability in its application, so advanced access is characterized by a model that guarantees greater accessibility to the patient. Based on this review, there is evidence to suggest that implementing AA models reduces ED utilization.

For realities in which there are public and universal healthcare systems, no relevant studies were found, so the results found were, for the most part, for patients insured by health plans in the United States and Canada. It is suggested that research related to the implementation of advanced access and its clinical outcomes be carried out in different health systems and, in particular, in countries with a public system of universal access.

## Figures and Tables

**Table 1:** PICO strategy structure.

Population	Patients followed in primary healthcare who seek care in the emergency department
Intervention	Advanced access or open access in PHC
Comparator	Traditional access
Outcomes	Reduction in demand for emergency services

**Table 2:** Assessment of quality of the cohort studies based on the Newcastle-Ottawa scale (NOS).

Author	Selection				Comparability	Outcome			
	Representativeness of the Exposed Cohort	Selection of the Non-Exposed Cohort	Ascertainment of Exposure	Demonstration that Outcome of Interest Was Not Present at Start of the Study	Comparability of Cohorts on the Basis of the Design or Analysis	Ascertainment of Outcome	Was Follow-Up Long Enough for Outcomes to Occur	Adequacy of Follow Up of Cohorts	Total
Cook [31]	*	*	*		**	*	*	*	8
Glass [34]	*	*		*	*	*	*	*	7
Yoon [32]				*	**	*	*	*	6

The scale provides a rating system ranging from 0 to 9 stars. * is equal to one star. ** is equal to two stars

**Table 3:** Quality assessment tools. (**A**) Quality assessment tool for before–after (pre–post) studies with no control group, (**B**) quality assessment tool for observational cohort and cross-sectional studies.

(A) Solberg et al., 2004 [35]	
Was the question or objective of the study clearly stated?	Yes
Were the eligibility/selection criteria for the study population prespecified and clearly described?	Yes
Were the participants in the study representative of those who would be eligible for the test/service/intervention in the general or clinical population of interest?	Yes
Were all eligible participants that met the prespecified entry criteria enrolled?	Yes
Was the sample size sufficiently large to provide confidence in the findings?	Yes
Was the test/service/intervention clearly described and performed consistently in the study population?	Yes
Were the outcome measures prespecified, clearly defined, valid, reliable, and assessed consistently across all study participants?	Yes
Were the people assessing the outcomes blinded to the participants’ exposures/interventions?	No
Was the loss to follow-up after baseline 20% or less? Were those lost to follow-up accounted for in the analysis?	Yes
Did the statistical methods examine changes in outcome measures from before to after the intervention? Were statistical tests conducted that provided *p* values for the pre-to-post changes?	Yes
Were outcome measures of interest taken multiple times before the intervention and multiple times after the intervention (i.e., did they use an interrupted time-series design)?	No
If the intervention was conducted at a group level (e.g., a whole hospital, a community, etc.) did the statistical analysis take into account the use of individual-level data to determine effects at the group level?	Yes
**(B) Hudec, Macdougall, et al., 2010 [33]**	
Was the research question or objective in this paper clearly stated?	Yes
Was the study population clearly specified and defined?	Yes
Was the participation rate of eligible persons at least 50%?	Yes
Was a sample size justification, power description, or variance and effect estimates provided?	Yes
For the analyses in this paper, were the exposure(s) of interest measured prior to the outcome(s) being measured?	Yes
Was the timeframe sufficient so that one could reasonably expect to see an association between exposure and outcome if it existed?	No
For exposures that can vary in amount or level, did the study examine different levels of the exposure as related to the outcome (e.g., categories of exposure, or exposure measured as a continuous variable)?	No
Were the exposure measures (independent variables) clearly defined, valid, reliable, and implemented consistently across all study participants?	No
Was the exposure(s) assessed more than once over time?	No
Were the outcome measures (dependent variables) clearly defined, valid, reliable, and implemented consistently across all study participants?	Yes
Were the outcome assessors blinded to the exposure status of participants?	No
Was loss to follow-up after baseline 20% or less?	Yes
Were key potential confounding variables measured and adjusted statistically for their impact on the relationship between exposure(s) and outcome(s)?	Yes

**Table 4:** Summary of rapid review of studies on advanced access.

Author	Study Description	Intervention	Outcome
Glass 2017 [34]	Trends in visits and costs were compared between the intervention group (n = 1211) and the control group (n = 542,162) for six types of visits, including urgency and emergency. In the intervention group, a medical center was opened in the industry, eliminating the need to travel to obtain care and guaranteeing advanced access. The data before and after the intervention were compared in the two groups.	Implementation of a PHC center at the workplace, ensuring same-day access and no need to travel. Visits were divided into six categories, including urgency and emergency.	Reduction in emergency visits in the intervention group compared to the control group (−43% vs. −5%, *p* < 0.001). There were no differences in emergency visits. It is noted that PHC visits increased by 43% in the intervention group and only 4% in the control group (*p* < 0.001).
Yoon 2015 [32]	Patients (n = 71,296) were identified from 22 VHA PHC clinics. Their use of services was obtained through VHA administrative systems, without individual patient identification. Same-day access was measured as the percentage of patients receiving care in PHC within less than 1 day of requesting care. The use of services was divided into 7 distinct groups.	Implementation, in 2010, of “Patient Aligned Care Teams”, focusing on improving access. Same-day access was defined as the percentage of patients receiving PHC within less than 1 day of request.	Clinics with low same-day access (<40% of patients receive same-day care) had more ED visits (*p* < 0.001). A 10% increase in patients receiving care the same day they requested it was associated with a 6% reduction in all-cause ED visits (*p* = 0.002).
Solberg 2004 [35]	Approximately 7000 patients with diabetes, 3800 with cardiovascular disease, and 6000 patients with depression were included in the study. These patients were identified between 1998 and 2001. Their service utilization and cost were obtained from health plan administrative systems. The types of visits to the health service were grouped into four groups. The data were comparatively evaluated pre-intervention (improved access in 2001) and after intervention.	Complete advanced access in January 2001. Visits to the health service were grouped into four groups (>1 emergency visit, >1 urgency visit, >1 hospital admission, and length of hospital stay greater than 3 days).	Emergency room visits were reduced by approximately one-third (*p* < 0.001), but the change was not statistically significant in ED visits (*p* = 0.68/*p* = 0.78/*p* = 0.15).
Cook 2020 [31]	205 primary care physicians, with at least 13 TNAA measurements per year. The TNAA for each professional was obtained from the period between January 2009 and January 2017. The enrolled population of each patient was linked to the use of all outpatient levels, including emergency services.	Assessment of in three groups: improvement, stabilization, or worsening of TNAA.	Providers who improved their TNAA over a 1-year period saw a reduction in ED visits by 78 visits per 1000 patients per year (1.5 × 52 wk—*p* < 0.05).
Hudec 2010 [33]	Semi-structured interviews were conducted with professionals from four teams (3 traditional access and 1 advanced access) and their reports were found, and reports on the use of emergency services were obtained from them. Economic indicators for the months of April, May, and June in the year of study and in the previous year were also evaluated.	Advanced access with results measured by self-reported questionnaire and costs.	In the interviews, the benefit of advanced access was cited as reducing the use of the emergency department. Based on economic indicators, a 28% reduction was observed in the triage of less urgent patients in the emergency department.

## Data Availability

The original research papers included in this rapid review are in the public domain. All data extracted in the rapid review are included in the tables of the manuscript.

## Abbreviations

The following abbreviations are used in this manuscript:

AA	Advanced access
ED	Emergency department
PHC	Primary healthcare
WHO	World Health Organization
TNAA	Third-next appointment available
NOS	Newcastle-Ottawa Scale (NOS)
